# Visual Estimation of Tricuspid Annular Plane Systolic Excursion by Emergency Medicine Clinicians

**DOI:** 10.5811/westjem.2020.5.46714

**Published:** 2020-07-10

**Authors:** Youyou Duanmu, Andrew J. Goldsmith, Patricia C. Henwood, Elke Platz, Janet E. Hoyler, Heidi H. Kimberly

**Affiliations:** * Stanford University School of Medicine, Department of Emergency Medicine, Palo Alto, California; † Harvard Medical School, Massachusetts General Hospital, Department of Emergency Medicine, Boston, Massachusetts; ‡ Thomas Jefferson University, Department of Emergency Medicine, Philadelphia, Pennsylvania; § Harvard Medical School, Brigham and Women’s Hospital, Department of Emergency Medicine, Brigham and Women’s Hospital, Boston, Massachusetts; ¶ 123Sonography, Cohasset, Massachusetts; || Newton Wellesley Hospital, Department of Emergency Medicine, Newton, Massachusetts

## Abstract

**Introduction:**

Tricuspid annular plane systolic excursion (TAPSE) is an established echocardiographic marker of right ventricular (RV) systolic function. The objective of this study was to evaluate whether emergency clinicians can visually estimate RV function using TAPSE in a set of video clips compared to a reference standard M-mode measurement.

**Methods:**

Emergency clinicians were shown a five-minute educational video on TAPSE. Participants then viewed 20 apical four-chamber point-of-care ultrasound (POCUS) echocardiography clips and recorded their estimate of TAPSE distance in centimeters (cm), as well as whether TAPSE was normal (>1.9 cm), borderline (1.5–1.9 cm), or abnormal (<1.5 cm). We calculated sensitivity, specificity, and overall accuracy of visual TAPSE categorization using M-mode measurement as the criterion standard. Participants also reported their comfort with assessing TAPSE on a five-point Likert scale before and after participation in the study.

**Results:**

Among 70 emergency clinicians, including 20 postgraduate year 1–4 residents, 22 attending physicians, and 28 physician assistants (PA), the pooled sensitivity and specificity for visual assessment of TAPSE was 88.6% (95% confidence interval, 85.4–91.7%) and 81.6% (95% CI, 78.2–84.4%), respectively. The sensitivity and specificity for the clips in which the measured TAPSE was <1.5 cm or >1.9 cm was 91.4% (95% CI, 88.4–94.3%) and 90.8% (95% CI, 87.7–93.9%), respectively. There was no significant difference in sensitivity (p = 0.27) or specificity (p = 0.55) between resident and attending physicians or between physicians and PAs (p = 0.17 and p = 0.81). Median self-reported comfort with TAPSE assessment increased from 1 (interquartile range [IQR] 1–2) to 3 (IQR 3–4) points after participation in the study.

**Conclusion:**

A wide range of emergency clinicians demonstrated fair accuracy for visual estimation of TAPSE on previously recorded POCUS echocardiography video clips. These findings should be considered hypothesis generating and warrant validation in larger, prospective studies.

## INTRODUCTION

Venous thromboembolism (VTE) including pulmonary embolism (PE) represents the third leading cause of vascular disease worldwide after myocardial infarction and stroke, with approximately 300,000 deaths each year from PE in the United States.^1^–^6^ The timely diagnosis of PE in the emergency department (ED) is critical to the management of a condition that carries significant morbidity and mortality.^6^

Evidence of right heart dilation or impaired function on point-of-care ultrasound (POCUS) are findings that can be associated with the presence of PE. POCUS is a required competency in emergency medicine, should be available in the ED setting, and can be rapidly performed at the bedside. Evidence of right ventricular dysfunction (RVD) on ultrasound is both a predictor of PE diagnosis as well as PE severity and clinical outcomes.^7^–^9^ One of the markers of RVD on ultrasound is reduced tricuspid annular plane systolic excursion (TAPSE).^10^ The measurement of TAPSE involves obtaining an apical four-chamber ultrasound view, placing the M-mode line at the lateral tricuspid valve annulus (where the valve leaflet attaches to the wall of the right ventricle), obtaining an M-mode tracing and measuring the height of the annulus movement during systole.^11^ Besides its utility in diagnosing PE, TAPSE has also been applied as a marker of pulmonary hypertension and, more recently, as a predictor for the development of cardiac tamponade in patients with malignant effusions.^12^,^13^ A prior study showed that investigators trained in echocardiography could visually estimate TAPSE as normal or abnormal with good agreement.^9^ However, to our knowledge, there is no prior literature examining whether emergency clinicians with a diverse level of training can accurately visually estimate TAPSE.

The primary aim of this study was to assess the sensitivity, specificity, and overall accuracy of emergency clinicians’ visual estimation of TAPSE as compared to standard M-mode measurements. Secondary aims were to evaluate the accuracy of visual estimation of TAPSE compared to visual estimation of right ventricle (RV) to left ventricle (LV) ratio, as well as the self-reported comfort level of emergency clinicians with TAPSE assessment after a brief training intervention.

## METHODS

The study was performed at an urban, university-affiliated, tertiary-care ED with a patient volume of approximately 70,000 per year. The institution has an emergency ultrasound division, emergency ultrasound fellows, and a resident ultrasound-training program. All attendings, residents, and mid-level clinicians in emergency medicine (EM) were eligible for enrollment in the study. The study was reviewed by the local institutional review board and determined to be exempt.

### Study Participants

We approached all emergency clinicians (including 65 attendings, 58 residents and 33 physician assistants [PA]) in the department for study participation. Participation was solicited by an email sent to all EM-trained attendings, EM residents, and ED PAs. Participation was voluntary and uncompensated. There were no exclusion criteria.

**Population Health Research Capsule**

What do we already know about this issue?

*Tricuspid annular plane systolic excursion (TAPSE) is a marker of right heart function that can be a predictor of the diagnosis and severity of pulmonary embolism*.

What was the research question?

Can emergency clinicians of diverse training levels accurately visually estimate TAPSE after brief training?

What was the major finding of the study?

*Emergency clinicians had fair accuracy for the visual estimation of TAPSE, which is hypothesis generating*.

How does this improve population health?

*Incorporating visual TAPSE assessment to point-of-care ultrasound techniques in the emergency department may improve the assessment of right heart dysfunction*.

### Study Design

Study participants were shown a five-minute educational video on TAPSE. The educational video was comprised of PowerPoint^TM^ slides with a concurrent audio recording outlining the concept of TAPSE and the definitions of normal and abnormal TAPSE, including ultrasound clips demonstrating examples of each. The video was produced and edited by the study authors. Participants then viewed 20 apical four-chamber ultrasound clips. Images were selected by a study investigator to have a wide range of TAPSE values spanning the normal, borderline, and abnormal categories. All images were obtained from our departmental POCUS database and had been reviewed by ultrasound fellowship trained division faculty. Images in the database were previously de-identified, and clinical data were therefore not available for review.

**Figure 1:**
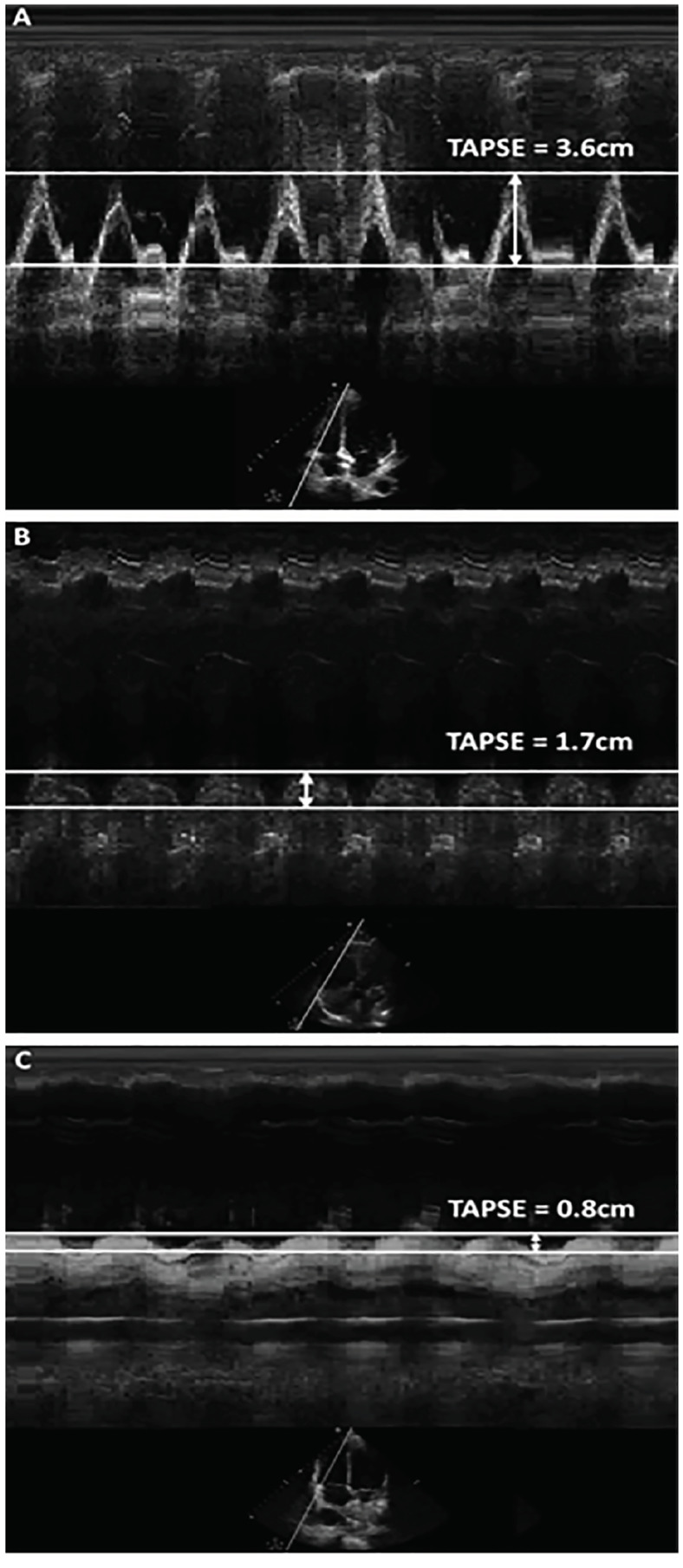
The measurement of tricuspid annular plane systolic excursion (TAPSE) from apical 4-chamber ultrasound clips by using the M.mode.ify open source software in normal (A), borderline (B), and abnormal (C) TAPSE. This technique was applied to determine the reference standard TAPSE measurement for each clip. M-mode images were not shown to the participants. *TAPSE*, tricuspid annular plane systolic excursion; *cm*, centimeter.

The TAPSE for each of the 20 apical four-chamber clips was measured by an ultrasound fellowship-trained emergency physician (EP) using the M.mode.ify application,^14^ an open-source software that generates an M-mode image from any B-mode ultrasound clip (Figure 1). The M.mode.ify application uploads a B-mode ultrasound clip and prompts the user to place an M-mode line. The application then crops and aligns still frames from the ultrasound clip corresponding to the location of the user-placed line in order to splice together an M-mode image. In this study, the M.mode.ify M-mode line was placed over the tricuspid annulus in the same manner as when measuring TAPSE on an ultrasound machine. The M.mode.ify TAPSE for each clip was measured twice. If there was disagreement, a second ultrasound fellowship-trained EP performed a third measurement for final agreement. Only three clips required a third measurement due to differences between the first two measurements of 0.1cm or 0.2cm.

The M.mode.ify M-mode measurement performed by an ultrasound fellowship-trained EP was considered the gold standard of TAPSE assessment for each clip. A measured TAPSE of 1.7 centimeters (cm) or greater was accepted as normal per the recommendations from the American Society of Echocardiography (ASE).^10^ The reference standard for the RV to LV size ratio estimate was calculated by a registered diagnostic medical sonographer using the measured ratio of the diameter of the right ventricle vs the left ventricle at the level of the mitral and tricuspid valves at end diastole.^15^

Clips were displayed in random order. Each clip was six seconds long and was played for three consecutive loops, for a total of 18 seconds per clip. While viewing each video clip, participants recorded their visual estimate of TAPSE distance in cms. They were informed of the current ASE guideline cutoff of ≥ 1.7 cm, as well as the study categorizations of normal TAPSE as (>1.9 cm), borderline as (1.5–1.9 cm) and abnormal as (<1.5 cm). We chose these three-category cutoff numbers during the design of this study as some studies on RV dysfunction have used the ASE cutoff of 1.7 cm while others have proposed using a normal TAPSE cutoff of >1.9 cm.^9^,^12^ In addition, participants were asked to note whether the RV:LV ratio appeared normal (<0.9), borderline (0.9–1.1), or abnormal (>1.1), with standard normal defined as an RV size less than a 1:1 size of the LV. These categorizations were chosen based on literature proposing RV:LV ratio cutoffs between 0.9 and 1.12 for predicting PE severity.^16^,^17^

Participants were asked how many previous TAPSE measurements they had performed. They were also asked to rate their comfort level with TAPSE assessment on a Likert scale from 1–5 with 1 indicating “not at all comfortable” with TAPSE and 5 indicating “very comfortable” with this concept, numbers 2 though 4 were not labeled. The Likert score was recorded both before and after the review of the educational video and the entire series of ultrasound clips. Enrollment for each subject took place in a single, in-person session.

### Statistical Analysis

We performed a sample size calculation according to the methods described by Buderer (1996).^18^ For an expected sensitivity of 95% and specificity of 86% (assuming that accuracy of visual estimation of RV function would be similar to visual estimation of LV function), a positive finding prevalence of 50%, acceptable precision of 10% and a significance level of 0.05, the desired sample size was 82 participants for adequate power in calculating sensitivity and specificity.^19^ Participants’ visual estimation of TAPSE distance in centimeters was used to stratify their assessment into the two-category classification of normal or abnormal (≥ 1.7 cm or < 1.7 cm) as well as the three-category classification of normal, borderline or abnormal (>1.9 cm, 1.5–1.9 cm, or <1.5 cm).

We calculated the sensitivity and specificity of participants’ two-category TAPSE assessment using ultrasound expert-performed M.mode.ify measurement of TAPSE as the reference standard. The overall accuracy of the participants’ three-category TAPSE estimate was also calculated. We calculated overall accuracy of the participants’ three-category RV:LV ratio assessment by using the RV:LV ratio measurement performed by a registered sonographer as the reference standard.

We used Mann-Whitney U test to compare sensitivity and specificity between training levels. Wilcoxon signed-rank test was used to evaluate TAPSE comfort level before and after the intervention. We considered a p value <0.05 statistically significant. Data was analyzed using Stata 14.2 (Stata Corporation, College Station, TX).

## RESULTS

A total of 70 emergency clinicians including 20 postgraduate year (PGY) 1–4 residents (7 PGY-1, 5 PGY-2, 5 PGY-3 and 3 PGY-4), 22 attending physicians, and 28 PAs participated in the study. While four (18%) of the attending physicians had previously completed an ultrasound fellowship, the majority of attendings (16, 73%) reported never previously having measured TAPSE. Half of the residents (10, 50%) and the majority of physician assistants (26, 93%) also reported no prior experience with TAPSE measurement. The range of TAPSE values in the 20 clips was 0.3–3.6 cm with 11 (55%) of the clips having abnormal TAPSE.

The pooled sensitivity and specificity for visual assessment of TAPSE was 88.6% (95% confidence interval [CI], 85.4–91.7%) and 81.6% (95% CI, 78.2–84.4%) respectively. The sensitivity and specificity for correct assessment of the clips (16/20) in which the measured TAPSE was not borderline (<1.5 cm or >1.9 cm) was 91.4% (95% CI, 88.4–94.3%) and 90.8% (95% CI, 87.7–93.9%), respectively.

The overall accuracy for the correct classification of TAPSE into the three categories of normal, borderline, and abnormal was 72.9% (95% CI, 70.4–75.3%). Table 1 shows the percent accuracy of TAPSE categorization within each of these three categories. The overall accuracy for correct categorization of non-borderline TAPSE into normal and abnormal was 91.2% (95% CI, 89.2–93.3%). The overall accuracy for the correct categorization of RV:LV ratio into the three categories of normal, borderline, and abnormal was 61.5% (95% CI, 59.6–63.5%). Table 2 shows the percent accuracy of RV:LV categorization within each of these three categories. The overall accuracy for the correct categorization of non-borderline RV:LV ratio into normal and abnormal was 71.4% (95% CI, 69.1–73.8%).

**Figure 2:**
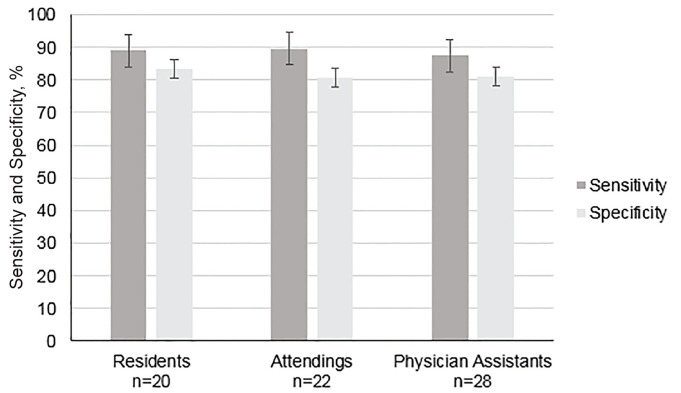
Comparison of sensitivity and specificity of tricuspid annular plane systolic excursion (TAPSE) visual estimation by training level.

There was no significant difference in the sensitivity (88.9% vs 89.7%, p = 0.27) or specificity (83.33% vs. 80.8%, p = 0.55) of visual TAPSE assessment between resident and attending physicians or between physicians and PAs (89.3% vs 87.4%, p = 0.17 for sensitivity and 82.0% vs 80.9%, p = 0.81 for specificity) (Figure 2). Median TAPSE assessment comfort score increased from 1 (interquartile range [IQR] 1–2) to 3 (IQR 3–4) after participation in the study among all participants.

## DISCUSSION

Assessment of RV function is an important aspect of the emergent evaluation of patients presenting with cardiopulmonary complaints, particularly when there is concern for PE. In this study, emergency clinicians of diverse training levels were able to categorize TAPSE as normal or abnormal by visual assessment with fair sensitivity and specificity when compared to TAPSE M-mode measurement, after review of a brief educational video. The overall significance of these findings is limited by a slightly smaller than targeted sample size, which may have led to insufficient power of the study’s results.

Although EPs have been shown to be able to use M-mode to measure TAPSE as a marker of RV systolic function, this measurement can be cumbersome for many users.^9^ It has been previously demonstrated that EPs can visually estimate left ventricular (LV) ejection fraction with good agreement as compared to cardiologists.^19^ If emergency clinicians could also estimate RV systolic function visually at the point-of-care without performing M-mode measurements, this could be a time efficient addition to the assessment of cardiac function the ED. We believe that our results provide early, hypothesis generating evidence that this may be the case.

In patients with PE, a finding of RV dysfunction as demonstrated by low TAPSE measurement has been associated with increased mortality, longer length of hospital stay, and the development of pulmonary hypertension.^7^,^8^ TAPSE has been shown to be the least user-dependent evaluation of RV dysfunction and has been shown to be a feasible measurement by emergency physicians.^9^,^20^ Prior literature also suggests that TAPSE is a sensitive marker for PE in patients with tachycardia or hypotension.^9^,^21^

In the current study, there was superior sensitivity and specificity for assessing normal vs. abnormal at the more extreme ranges of TAPSE as compared to the overall sensitivity and specificity of TAPSE assessment as normal or abnormal. Patients with severely reduced TAPSE from conditions such as a massive or sub-massive PE would likely require the most time-sensitive clinical interventions.^21^ Therefore, the ability to correctly identify significantly impaired RV dysfunction with a quick visual assessment may represent a valuable clinical application. Our data implies that patients with severely reduced TAPSE are more accurately identified than those with borderline RV dysfunction.

Interestingly, visual estimation of RV to LV ratio had only fair accuracy across all participants in our cohort, despite this being the accepted POCUS method for evaluating for right ventricular dysfunction, and which is taught to emergency clinicians at our institution.^22^,^23^ Even excluding borderline RV size clips, the overall accuracy of correct categorization of RV as normal or abnormal was only 71.4%. This may have been due to the fact that RV size may be more difficult to estimate or due to the fact that the visual estimation of RV:LV size was not included in the educational video but assumed to be known by the study participants. The overall accuracy of correct categorization of non-borderline TAPSE was 91.2%. Paczyńska and colleagues demonstrated recently that TAPSE was a better predictor of adverse outcomes in patients with acute PE than RV to LV ratio.^24^ Our study suggests that visual TAPSE assessment could be a more accessible and accurate aspect of the POCUS assessment of right heart function than relative RV size for emergency clinicians in the acute care setting. Whether dedicated training in the assessment of RV size could improve emerency clinicians’ accuracy in this measure warrants further investigation.

A prior study by Daley et al demonstrated that a small sample of three ultrasound fellowship-trained EM attendings, four ultrasound fellows as well as an EM resident and medical student with several weeks of training in TAPSE measurement could visually estimate its value with good agreement.^9^ In comparison, the majority of participants in our study had never previously measured TAPSE. To our knowledge, this is the first study to examine the visual estimation of TAPSE by non-physician clinicians. Advance practice providers (APP) such as PAs and nurse practitioners have become increasing prevalent in EM, and the use of POCUS by APPs is a growing aspect of their practice.^25^ Visual TAPSE assessment was easily learned by those who had little or no prior exposure to the concept of TAPSE. There was no significant difference in the overall sensitivity or specificity of visual TAPSE assessment between junior and senior residents, residents, and attendings, or physicians of all levels of training and PAs. Although a very brief training video appeared to increase participants’ confidence in visual TAPSE assessment, more extensive education is likely required for this skill to be applied in clinical practice. Further study as to whether diverse groups of emergency clinicians in different types of hospital settings can visually assess TAPSE, and whether this correlates to clinically significant disease processes is warranted.

## LIMITATIONS

This study was limited by smaller than targeted sample size due to the lack of sufficient volunteers, which may have led to insufficient power of the study’s results. Enrollment took place at a single academic institution with a robust ultrasound training program, which may limit generalizability to other institutions and practice settings. Emergency clinicians volunteered to participate in the study, which may have introduced a selection bias. The clips were selected from an ultrasound image database but may not have been representative of those encountered in clinical practice. Furthermore, because the clips were selected from a de-identified database, the clinical information for the patients (ie, whether they had a diagnosis of PE or other right heart pathology) is unknown. Results could vary when clinicians both obtain the POCUS images as well as estimate the TAPSE in clinical practice. Finally, description of the M.mode.ify software has been published in peer-reviewed literature, but independent validation of how well it corresponds to ultrasound machine M-mode measurements has not been established.

## CONCLUSION

The ability to estimate RV systolic function visually at the point of care could represent a valuable addition to the assessment of cardiac function in the ED. This study suggests that visual TAPSE estimate is easy to learn and may be a feasible surrogate for measurements, particularly in non-borderline cases.

## Figures and Tables

**Table 1:** Percentage accuracy of visual categorization of tricuspid annular plane systolic excursion (TAPSE) into the categories of normal (>1.9 centimeters [cm]), borderline (1.5–1.9 cm) and abnormal (<1.5) as compared to measured TAPSE.

	Measured TAPSE Normal	Measured TAPSE Borderline	Measured TAPSE Abnormal
Visual TAPSE Normal	83%	4%	2%
Visual TAPSE Borderline	16%	42%	19%
Visual TAPSE Abnormal	2%	54%	79%

Darker shading indicates more accurate categorization.

**Table 2:** Percentage accuracy of visual categorization of right ventricle (RV) to left ventricle (LV) ratio into the categories of normal (<0.9), borderline (0.9–1.1), and abnormal (>1.1) as compared to measured RV:LV ratio.

	Measured RV:LV Ratio Normal	Measured RV:LV Ratio Borderline	Measured RV:LV Ratio Abnormal
Visual RV:LV Ratio Normal	64%	35%	1%
Visual RV:LV Ratio Borderline	28%	32%	19%
Visual RV:LV Ratio Abnormal	8%	34%	80%

Darker shading indicates more accurate categorization.
